# Implementation of a model for identifying Essentially Derived Varieties in vegetatively propagated *Calluna vulgaris *varieties

**DOI:** 10.1186/1471-2156-9-56

**Published:** 2008-08-20

**Authors:** Thomas Borchert, Joerg Krueger, Annette Hohe

**Affiliations:** 1 Heidepflanzen Peter de Winkel, Douvenberg 34, 47547 Goch, Germany; 2 Institute of Vegetable and Ornamental Crops (IGZ), Dept. Plant Propagation, Kuehnhaeuser Str. 101, 99189 Erfurt, Germany

## Abstract

**Background:**

Variety protection is of high relevance for the horticultural community and juridical cases have become more frequent in a globalized economy due to essential derivation of varieties. This applies equally to *Calluna vulgaris*, a vegetatively propagated species from the *Ericaceae *family that belongs to the top-selling pot plants in Europe. We therefore analyzed the genetic diversity of 74 selected varieties and genotypes of *C. vulgaris *and 3 of *Erica *spp. by means of RAPD and iSSR fingerprinting using 168 mono- and polymorphisms. The same data set was utilized to generate a system to reliably identify Essentially Derived Varieties (EDVs) in *C. vulgaris*, which was adapted from a method suggested for lettuce and barley. This system was developed, validated and used for selected tests of interest in *C. vulgaris*.

**Results:**

As expected following personal communications with breeders, a very small genetic diversity became evident within *C. vulgaris *when investigated using our molecular methods. Thus, a dendrogram-based assay to detect Essentially Derived Varieties in this species is not suitable, although varieties are propagated vegetatively. In contrast, the system applied in lettuce, which itself applies pairwise comparisons using appropriate reference sets, proved functional with this species.

**Conclusion:**

The narrow gene pool detected in *C. vulgaris *may be the genetic basis for juridical conflicts between breeders. We successfully tested a methodology for identification of Essentially Derived Varieties in highly identical *C. vulgaris *genotypes and recommend this for future proof of essential derivation in *C. vulgaris *and other vegetatively propagated crops.

## Background

*Calluna vulgaris *L. (Hull.), an exclusive species within the genus *Calluna*, has increased its economic weight, and not only within the German horticultural industry over the last few decades: In 2005 almost 100 million plants were produced in Germany, of which about 30% were exported to other European countries [1] where the demand is also still increasing. Although merely a handful of breeders are commercially active in breeding *C. vulgaris*, more than 300 varieties now exist, which are or have been protected at the Bundessortenamt, Hannover (BSA) [2] and/or the Community Plant Variety Office (CPVO), Angers, France [3]. More than 50% of applications for variety protection at the CPVO date from 2003 or later, which supports the argument of the increasing importance of *C. vulgaris*.

**Figure 1:**
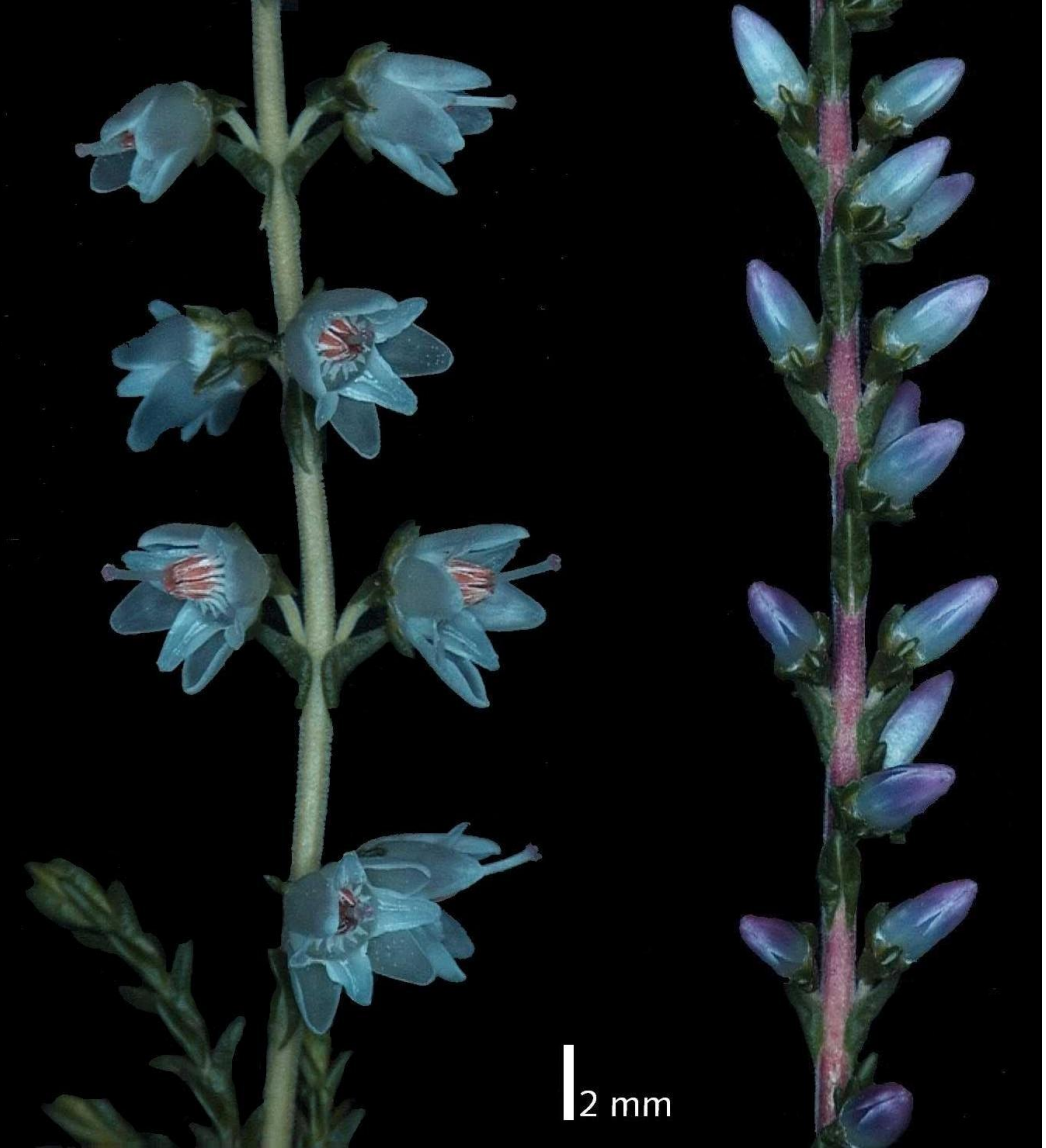
**Shoots of two *C. vulgaris *genotypes representing the main inflorescence types**. left: normal ('White Mite'), right: bud ('Anneliese').

Breeding efforts in *C. vulgaris *primarily aim at a special type of its inflorescence, the so-called bud flowers (Fig. 1). Flowers of these plants do not open during the entire reproduction phase from August to December which makes them appear visually attractive for a long period of time when not many other flowering ornamental outdoor plants are available in the northern hemisphere. This phenotype is closely linked with and possibly caused by a lack of anthers. This connection, in turn, has a severe impact on breeding methods because interesting bud-flowering genotypes are only applicable as the female parent in crossings. In addition, there is only sparse information and hypotheses available concerning the inheritance of this trait. Therefore – and since *C. vulgaris *is a vegetatively propagated crop – breeding in *C. vulgaris *over the past few decades was to a large extent performed by selection of spontaneous mutations, rather than by systematic crossings (personal communications with breeders). The actual variety composition in Europe offers a mixture of normal flowering and bud flowering types (state: 01/2008) with main focus on the latter (~85%). Some special forms (e.g. 'Radnor' with filled flowers or 'Peace' as a multi-bracteate type) are present as well. However, due to the problems described above, the actual gene pool used in breeding of *C. vulgaris *is presumably quite narrow.

Therefore, in this study the genetic diversity within the species *C. vulgaris *was examined with molecular DNA techniques, comprising a selection of 64 economic important and partially still-protected varieties from Germany, including varieties from other European countries and the USA, 5 genotypes resulting from crossings, as well as a selection of 5 wild plants of different origin. Moreover, 3 different genotypes of *Erica *spp. were included in this study as an anticipated outgroup [see Additional file 1].

In the case of *C. vulgaris*, variety protection assessments as executed by the CPVO and as described in the Protocol for Distinctness, Uniformity and Stability Tests for *Calluna *L. (Hull.), LING, Scots Heather (CPVO-TP/94/Final of 06/11/2003), comprises 22 phenotypic traits in total but only 18 traits for bud-flowering varieties, which are tested in 2 flowering seasons with 30 plants (replications). Herein, problems arise from continually increasing applications for protection of bud-flowering genotypes, from their partial identicalness in many of these traits and from the subjectiveness that is inherent in the measurement of phenotypic traits. Moreover, breeding of bud flowering types requires backcrossing, which is – in contrast to mutant selection or 'cosmetic breeding' – 'true breeding', but which also contributes to the narrow gene pool. Previously, these drawbacks led to some juridical disputes in the field of variety derivation in *C. vulgaris *in Germany.

The problem of variety derivation and the need for an appropriate protection system was already identified decades ago and is especially pressing in the context of global marketing. In Europe, the Act of Convention from 1991 followed on from a Convention on the Protection of Plant Varieties [4] and first introduced the term 'Plant Breeder's Rights'. Today it is acknowledged by 65 member states (, state: 01/2008). Variety protection in these member states is based on DUS-tests (distinctness, stability, uniformity: see above). Despite increasing testing efforts, these tests remain sketchy since the investigated traits may be influenced by several factors, e.g. environmental changes, and are evaluated by subjective ratings so that molecular markers have become a desirable tool [5]. But also the so-called 'fingerprinting techniques' – although widely recommended as a supplement to phenotypic tests e.g. by [6] – entail a number of problems, since only a random sample i.e. a subset of the genome, can be examined. Therefore, any statistical method applied to this problem has to be able to maintain a delicate balance in order to avoid excessive identification of false positives on the one hand, as well as false negatives on the other [6-8].

Several case studies have been recently published in the context of ED-conflicts, however, for the most part these do not concern vegetatively propagated species, since it is generally assumed that variation in such varieties does not occur, which would allow clear-cut molecular genotyping. [9] tested essential derivation in various vegetatively propagated ornamentals (*Rhododendron*, *Rosa*, *Phalaenopsis*) by AFLP-genotyping and constructing UPGMA-dendrograms. However, these investigations relied on the assumption of total genetic stability within vegetatively propagated varieties and therefore dispensed with any statistical analysis. From our point of view, this is not appropriate for all vegetatively propagated species, because – for example with *Calluna *– phenotypic variations (sports) are well-known and are based on genotypic variation. From these experiences we support EDV-identifying systems with respect to statistical validation as with the one introduced by [10] for lettuce and barley [11], which is based on the definition of a minimum distance (threshold) for distinctness. Such procedures are necessary since proving identity is more difficult than proving distinction with molecular markers [12]. Lettuce is a self-fertilizer and consequently genetic variation within varieties can be expected to be very low. Moreover total variation between today's cultivars should be somewhat reduced due to an intensive breeding history. For this reason, [11] suggested that ED-conflicts should not be analyzed through the construction of a dendrogram visualizing hypothetical kinship relations, but instead by the examination of all pairwise genetic distances within an appropriate reference population, and then comparing these results to the distance between actual varieties in question.

As a result, another aim of our study, drawing on the publication by [11], was to implement a comparable concept of identification of EDVs in *C. vulgaris *based on molecular data resulting from RAPD and iSSR techniques. Our system proposal is critically evaluated with regard to essential premises e.g. variation and stability [13], its success in *C. vulgaris*, and its practicability in the future.

The results presented here were obtained during a BMWi-(Federal Ministry of Economics and Technology) funded cooperation between the IGZ and a German breeding company (Heidepflanzen Peter de Winkel, ). Thus, variety denotation is ciphered in cases where the breeder's interests may be affected.

## Results

### Estimation of genetic diversity and kinship relations within *C. vulgaris*

**Figure 2:**
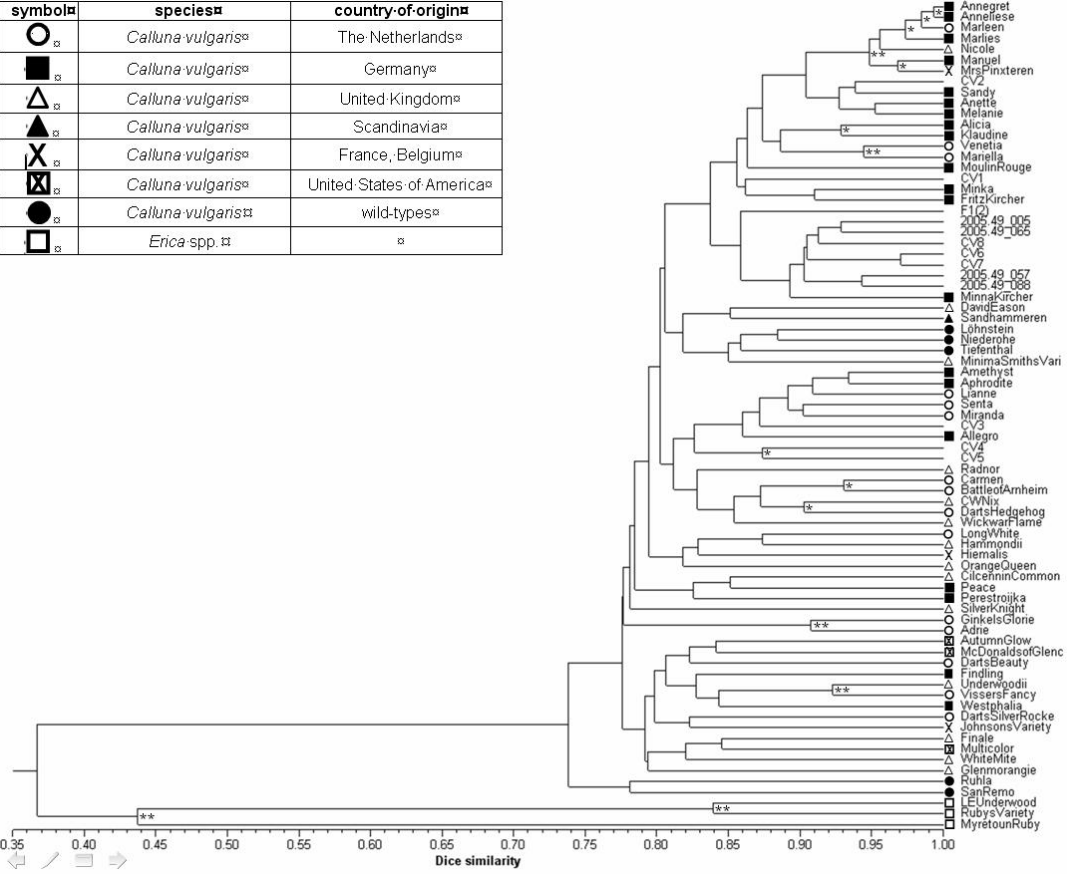
**Dendrogram consisting of 74 *C. vulgaris *and 3 *Erica *spp. genotypes**. Constructed from 168 mono- and polymorphisms amplified from 13 RAPD and 5 iSSR-primers and based on the Dice/Nei and Li coefficient with subsequent UPGMA-clustering. Nodes with strong support (> 85%) by bootstrapping (n = 10.000, PHYLIP) are marked with **, moderately supported groups (50% – 85%) are marked with *, varieties of interest for the involved company are ciphered by CV# where # is replaced by increasing numbers. Variety encryption is known to the authors and the company, respectively. For purposes of clarity and according to their regional provenance, genotypes have been classified by symbols as indicated.

Using RAPD- and iSSR-techniques, we achieved a total of 129 (RAPD) and 39 (iSSR) distinguishable and reproducible bands. This corresponds to 9.9 bands/RAPD primer and 7.8 bands/iSSR primer. The combined results of RAPD and iSSR studies are shown in the dendrogram in Fig. 2. While the three *Erica *genera do cluster as an outgroup, all tested genotypes from the *Calluna *species cluster to the right of one node. Interestingly, the wild-types from Thuringia (Ruhla) and from the Italian Alps (San Remo) cluster as an additional outgroup within the *Calluna *species while the other wild-types available (Löhnstein, Niederohe, Tiefenthal, all from the Lüneburger Heide in Germany) are grouped within the rest of the *Calluna *genotypes.

The statistical significance of our data was investigated with the resampling method of bootstrapping as initially described by [14] using the software Winboot and n = 10,000 replications. Those few nodes with moderate support (50% < p < 85%), as well as strong support (p ≥ 85%), which appeared both in the NTSYSpc-constructed tree as well as in the majority-rule consensus tree of Winboot, are marked with * and **, respectively, in Fig. 2. The linked genotypes to the right of these nodes may be considered to be linked in real kinship. Despite the high number of analyzed bands, all other linkages are statistically unconfirmable within the present data set.

### Identifying EDVs in *C. vulgaris*

Due to former juridical conflicts concerning property rights of varieties in the genus *Calluna *we endeavored to develop a reliable statistical system for identifying EDVs in this species based on the results from the first part of our study. Since the dendrogram analysis did not support statistically significant decisions on kinship relations and probably would not do so even after analysis with a clearly expanded data set, we decided to implement a method based on a procedure published by [11] for similar analyses in lettuce. We therefore created appropriate Reference Sets of 25 varieties (Table 1) for each pair of tested genotypes (Test Set) in question and then computed primer-wise and pair-wise similarity values within each set. The Test Sets were chosen to represent non-ambiguous EDV or clear non-EDV cases for proof of concept, as well as several cases of interest in *Calluna *(Table 2). This non-ambiguousness was derived from personal communications with the involved breeding company in case of the EDV-pair. The test of a BC_1 _against the parents as a clear non-EDV case was performed with our own crossings.

**Table 1:** Identifying essential derivation in *C. vulgaris*.

	**Reference Sets**	
	**Set A**	**Set B**

**1**	Niederohe	'Sandy'
**2**	San Remo	'Annegret'
**3**	'Adrie'	
**4**	'Allegro'	
**5**	'Boskoop'	
**6**	'Carmen'	
**7**	'C. W. Nix'	
**8**	'Dark Beauty'	
**9**	'Findling'	
**10**	'Glenmorangie'	
**11**	'Johnson's Variety'	
**12**	'Long White'	
**13**	'Mariella'	
**14**	'Marlies'	
**15**	'McDonalds of Glencoe'	
**16**	'Minima Smith's Variety'	
**17**	'Mrs. Pinxteren'	
**18**	'Multicolor'	
**19**	'Orange Queen'	
**20**	'Peace'	
**21**	'Radnor'	
**22**	'Sandhammeren'	
**23**	'Silver Knight'	
**24**	'Underwoodii'	
**25**	'Wickwar Flame'	

Identifying essential derivation in *C. vulgaris*: Reference-Sets A and B chosen for the Test-Sets. Set B differs from A only in the exchange of two wild-type genotypes (Niederohe, San Remo) versus 2 varieties ('Sandy', 'Annegret') as indicated by summarizing both columns of Set A and B for the residual 23 varieties.

**Table 2:** Identifying essential derivation in *C. vulgaris*.

**#**	**Test-Sets**		**selection criteria**	**Reference Set**	**Hypothesis**	**Result**
1	'Maria'	*Maria Hell*	Maria Hell = known sport of 'Maria' according to information from a breeder	A	yes	yes
2	'Maria'	BC_1_-individual	progeny testing	A	no	no
3	'Roter Oktober'	BC_1_-individual	progeny testing	A	no	no
4	'Melanie	'Anette'	'Anette' = sport of 'Melanie' according to information given by BSA doc	A	yes	yes
5	'Melanie'	'Sandy'	'Sandy' = sport of 'Melanie' according to information given by BSA doc	A	yes	yes
6	'Annegret'	'Anneliese'	'Anneliese' = sport of 'Annegret' according to information given by BSA doc	A	Yes	yes
7	'Fritz Kircher'	CV7	re-testing results from former investigations	A	yes	no
8	'Karla'	'Venetia'	similar cultivars from different breeders	A	no	no
9	'Minka'	'Miranda'	similar cultivars from different breeders	A	no	no
10	SanRemo	Ruhla	wild-type testing	B	no	no
11	Niederohe	Löhnstein	wild-type testing	B	no	no
12	Niederohe	SanRemo	wild-type testing	B	no	no

Test Sets and their criteria for selection, the applied Reference Set and our initial hypothesis with regard to whether or not ED was to be expected. The first three tests were used as proof of concept, meaning consistency of hypothesis and validation of the eligibility of the method; tests 4–12 are true testings.

**Figure 3:**
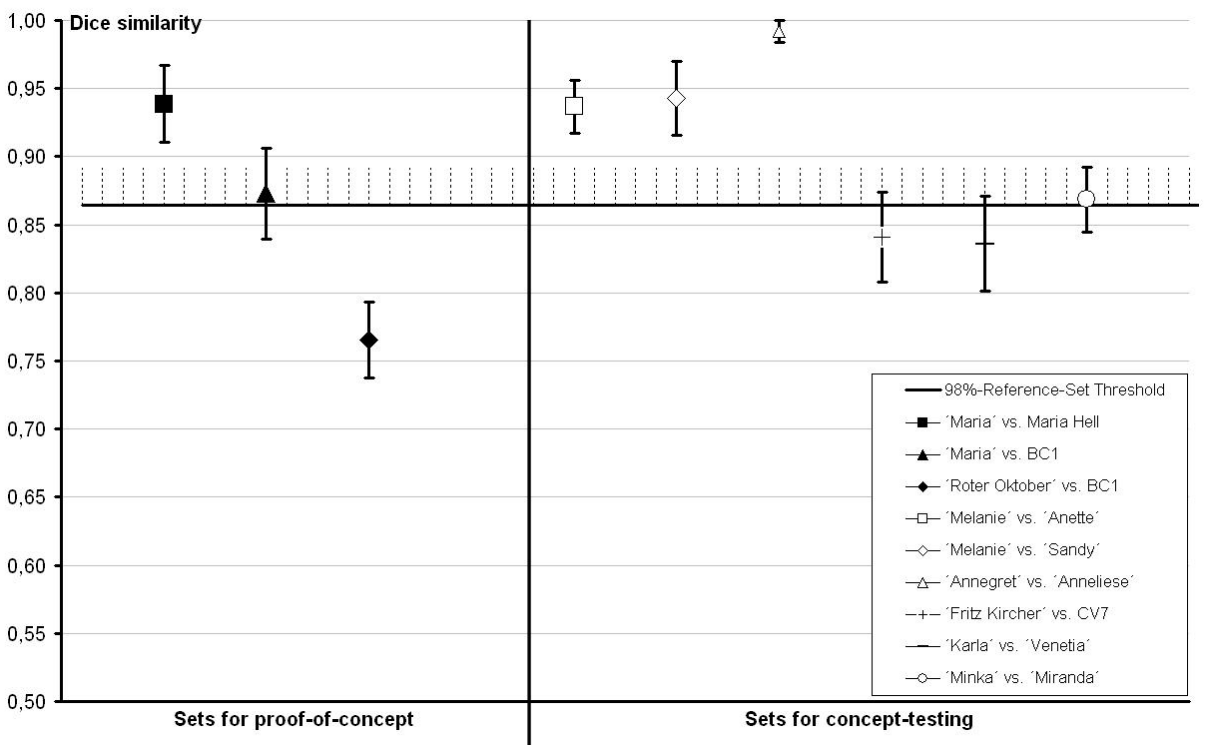
**Identifying essential derivation in *C. vulgaris *I**. Validation of the method using three sets for proof-of-concept: One set of a known essentially derived variety pair and two sets of genotypes involved in backcrossing, marked by black symbols. Additionally six pairs of varieties of interest have been tested against the chosen threshold of 0.865 Dice similarity value, which was derived from Reference Set A.

After extensive testing we selected a threshold provided by the highest Dice value of the 98% lowest values of all pairwise comparisons within the reference set (Fig. 3). This threshold was chosen in order to prevent the BC_1 _individual from being categorized as essentially derived from the backcross parent which constitutes an essential prerequisite for validation of our test since backcrossing is the normal breeding system in bud-flowering *Calluna*. The 98% thresholds in both Reference Sets differ due to the necessary adjustment of the reference set according to the test in question (exchange of wild-type genotypes against varieties from the upper cluster of the dendrogram): 98%-Set A: 0.865 Dice similarity value, 98%-Set B: 0.893 Dice similarity value.

For proof of concept we tested, on the one hand, one pair of individuals ('Maria' and *Maria Hell*), from which it was known that the latter was derived from the first one. On the other hand, an individual from a backcross progeny was tested against both parents, which should result in the categorization of being non-derived. As expected, the first result was positive and the second one negative, using the threshold as given above (Fig. 3). Moreover, similarity between the BC_1 _individual and the backcross parent was clearly higher than between the BC_1 _individual and the second parent. The Dice value of the comparison with the backcross parent was actually slightly above the threshold; however, overlapping error bars indicated that the similarity was nevertheless not sufficiently high for these two genotypes to be categorized as essentially derived.

**Figure 4:**
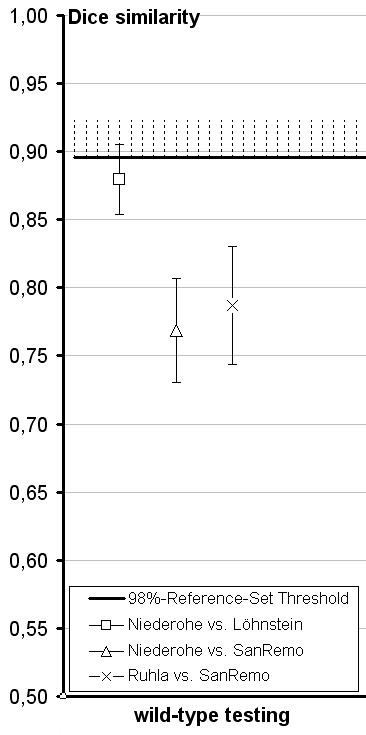
**Identifying essential derivation in *C. vulgaris *II**. Test of three pairs of wild types of different origin using Reference Set B (0.893 Dice similarity value).

Regarding the 'true tests', the results were negative for several pairs of morphologically similar cultivars from different breeders (Fig. 3), as well as for wild genotypes of different origin (Fig. 4). In contrast, when testing the cultivars 'Melanie' and 'Anette', their genetic similarity was found to exceed the threshold, thus confirming the public data supplied by the BSA according to which 'Anette' is a sport of 'Melanie'. This was also confirmed for 'Melanie' vs. 'Sandy' and 'Annegret' vs. 'Anneliese' (Fig. 3).

The last test concerned a pair of cultivars ('Fritz Kircher' vs. CV7) which have in a former, non-public study been characterized as being essentially derived from one another using dendrogram analysis. In our investigation, however, their genetic similarity is lower than the threshold, thus clearly indicating an absence of essential derivation.

## Discussion

Until now, molecular data on genetic diversity within the species *C. vulgaris *was only available for regionally restricted wild-type populations [15-17], not for varieties used in commercial breeding. An actual and urgent necessity for a comprehensive study in *C. vulgaris *can be deduced from several points: the number of applications for variety protection is currently increasing considerably, whereas the information given in the registration schedules is at least occasionally unreliable or equivocal (e.g. a bud flowering variety is said to be the result of selfing of another bud-flowering genotype, which is biologically impossible due to the total loss of anthers in bud-flowering genotypes). This leads to an ambiguous situation with regard to variety derivation. Additionally, molecular data are needed for concerted breeding works and the elimination of coincidence in this process.

Since it is technically simple to accomplish and requires no a priori sequence information, iSSR- and RAPD-PCR [18-20] are widely used techniques in different species; but RAPDs in particular may be 'considered the practice of PCR without a clue' [21]. All the same, both techniques provide a uniformly distributed amplification of DNA fragments throughout the genome of eukaryotic organisms due to the nature of their origin, and were shown to be an adequate molecular tool for studying DNA polymorphisms (e.g. [22,23]). The same was true for our investigations as we observed very robust inner-laboratory reproducibility: here, a value as low as 0.46% of missing data within the 77 × 168 similarity matrix was achieved.

The dendrogram resulting from the combined computation of both RAPD and iSSR banding patterns showed a low genetic variability within the species *C. vulgaris*: almost all tested varieties and genotypes are grouped at a Dice/Nei & Li similarity value of 0.80, or even higher. This confirmed our hypothesis of a narrow gene pool, which was expected by the breeding experiences and methods applied of the participating company (personal communications) and its competitors. Moreover, one has to bear in mind that *C. vulgaris *is the only species within the genus *Calluna *and that crossing with other genera of the *Ericaceae *is thus impossible, thereby assisting in the conservation in nature, too, of a slender genetic diversity. We esteem the clear discrimination between *Erica *and *Calluna *as one argument of reassurance for our methodological approach and consider the dendrogram to be unbiased in the sense of an essential prerequisite for picturing genetic data [11]. The fact that wild type genotypes from the Lüneburger Heide are grouped this near to economically important varieties is another piece of evidence for our line of argument in respect of a significantly narrow gene pool in *C. vulgaris*. In addition, to our knowledge, breeding in *C. vulgaris *began in exactly this area of Germany by collecting incidentally originated bud-flowering genotypes. Our results might thus confirm this hypothesis, especially since the wild types from Thuringia and the Italian Alps do not cluster within this group.

Another interesting feature of the resulting dendrogram is that the data were insufficient to support more than the few marked nodes (marked with * or ** in Fig. 2) as statistically significant. However, we do not consider the amount of bands i.e. mono-/polymorphisms from our data as generally too sparse, since [24] showed that an estimation of diversity within one population using approx. 200 dominant (i.e. AFLP) markers is as efficient as using 50 codominant (i.e. microsatellite) markers. Therefore, it is our suspicion, that the dendrogram method is not suitable for EDV identification in species with narrow gene pools.

ED issues arise for varieties that successfully passed DUS testing. An EDV is (i) predominantly derived from an initial variety, (ii) clearly distinguishable from it and except for these differences (iii) conforms to the initial variety in the expression of essential characteristics [5]. We consider it to be of paramount importance to apply a well adjusted system for identification of these EDVs for each species, and in our case for *C. vulgaris*, since the range of similarities presented in Fig. 2 proved the hypothesis of some breeders that the economically important varieties (and the genus *Calluna *in general) are closely related and thereby may readily lead to ED disputes, as has already been the case in the past.

As explained above, construction of a dendrogram proved to be no satisfactory tool for EDV identification in *C. vulgaris *– contrary to the results obtained for other vegetatively propagated species presented by [9] for *Phalaenopsis, Rosa *and *Rhododendron*. Another example is given by [25]. Using AFLPs, they proved that *Rosa × hybrida *original varieties are not more closely linked than 0.80 Jaccard's index. In contrast, the genetic similarities in so-called mutant groups were always higher than 0.96 (but not 1.0). Their dendrogram assay is therefore correctly rated as a suitable method to unambiguously distinguish rose EDVs from their initial variety. In addition, the detection of polymorphisms between sports and the original variety may be considered somewhat coincidental since molecular markers only cover a small portion of the target organism's genome. [26] demonstrated, that in cut roses RAPD-polymorphisms between a variety and its sports did occur in two varieties, but were not reproducible. Using AFLPs the authors were even able to amplify stable polymorphisms in sports of another variety. However, they were still able to distinguish vegetatively and sexually propagated progenies, since amplification in seedlings constantly resulted in a higher number of polymorphisms.

We ascribe our differing results to the coincidence of two phenomena in *C. vulgaris*. First, stable genetic conditions – which could be reasonably anticipated for vegetatively propagated species – are worthy of discussion in the context of *Calluna*, since the phenomenon of sport/reversion (a type of somatic mutation) is well-known by breeders.

Moreover, the very narrow gene pool in *C. vulgaris *gives rise to high genetic similarities, even if a new variety was obtained through crossing, due to the fact that even quite different individual plants, e.g. a wild type from the Lüneburger Heide and a bud flowering variety, show a considerable proportion of monomorphic bands in RAPD and iSSR analyses. Such lack of genetic diversity is our main reason for focusing on a system for EDV-identification involving a reference-set, as this is the important difference to e.g. the rose cases mentioned above: even in *Rosa × hybrida *more than 10,000 varieties exist, resulting from some 150 years of breeding efforts [25], and they are still clearly distinguishable. The opposite situation is, in fact, the result of the differing breeding methods applied in *C. vulgaris*: breeding for a common phenotype (bud-flowering) and repeated back-crossing are generally accepted reasons that promote the development of narrow gene pools [25].

By working with a system similar to that described for lettuce and barley by [11], we were successful in both, identifying well-known essentially derived genotypes as well as discriminating between a genotype resulting from backcrossing and its parents (Fig. 3). We considered these results as a proof of concept for our method and additionally analyzed other test-sets whose information of origin we regarded to be unreliable, questionable or simply of interest. Here, information on variety derivation was primarily confirmed by our method as outlined in table 2. Moreover, the system discriminated phenotypically similar varieties from different breeders as well as wild genotypes of different origin, thus also confirming the hypotheses.

## Conclusion

As a result of these findings, we would like to suggest the outlined method as an appropriate system for EDV-testing in *C. vulgaris*. Applicability to other vegetatively propagated crops should be tested, as well as the combined use of 'fixed' and 'random/unmapped markers' as suggested by [11]. Moreover, we recommend the inclusion of at least three independent gDNA isolations of different individuals per genotype, since inner-varietal identity cannot be presumed and is hard to verify, even in vegetatively propagated crops.

## Methods

### DNA techniques: isolation of genomic DNA (gDNA)

gDNA of *C. vulgaris *genotypes was isolated according to [27]. About 200 mg young leaf tissue (stored over night and frozen in liquid nitrogen) was homogenized in 2 ml tubes in a mixer mill (MM301, Retsch) using 2 stainless steal balls (Ø = 5 mm). The tissue was resuspended in buffer A (50 mM Tris-HCl pH 8.0, 5 mM EDTA, 350 mM sorbitol, 1% β-mercaptoethanole, 10% PEG-6000) and centrifuged for 1 min at 4°C and 8,000 rpm (Sigma 3K30, rotor-no. 12148). The resulting pellet was again resuspended in buffer B (50 mM Tris-HCl pH 8.0, 5 mM EDTA, 350 mM sorbitol, 1% β-mercaptoethanole, 1% sodiumsarcosyle, 0.1% CTAB, 710 mM NaCl) and incubated for 30 min at 60°C. After adding 0.8 volumes chloroform-isoamyl alcohol 24:1, the samples were centrifuged for 15 min at 4°C at 15,300 rpm. The supernatant was transferred to a new 2 ml reaction tube and incubated at -20°C for 30 min after adding 0.75 volumes isopropanole. After centrifugation (5 min at 4°C at 5,000 rpm) the pellet was washed with 70% ethanol, air-dried and resuspended in 500 μl TE buffer. To each sample, 1 ng RNAse (Carl Roth GmbH) was added, followed by incubation for 15 min at 37°C. Subsequently, phenol-chloroform extraction step was performed twice and the resulting supernatant containing purified gDNA was pelleted at -20°C for 60 min after addition of 0.1 volumes 3 M sodium acetate and 0.75 volumes isopropanole. The precipitated gDNA was washed twice with 70% ethanol, air-dried and resuspended in 100 μl TE. Long-time storage was achieved at -20°C.

### DNA techniques: PCR amplification and electrophoresis

Amplifications of RAPD-fragments generated from random decamer primers (Carl Roth GmbH) were performed in a Primus 96 advanced thermocycler (peqlab GmbH) using the following protocol: 5 min at 95°C, [1 min at 95°C, 1 min at 35°C, 1 min at 72°C]_35×_, 10 min at 72°C. The reaction mixture for a total volume of 25 μl contained 1× reaction buffer, 2.5 mM MgCl_2_, 1 U Taq.-DNA Polymerase (recombinant, Invitrogen), 0.2 mM of each dNTP (Invitrogen), 0.5 μM primer (Carl Roth GmbH, MWG Biotech AG), 10 ng gDNA and the adequate amount of sterile deionized H_2_O.

Amplification of iSSR-Fragments was performed following the same protocol as described for RAPDs, with the altered annealing temperatures according to primer length. Table 3 provides an overview of primers used in this study; these were chosen after screening 60 decamer primers for reproducibility. Decamer primers were obtained as random primer kits from Carl Roth GmbH; iSSR primers were synthesized by MWG Biotech AG. iSSR primers given in Table 3 were chosen by referring to the common di- and trinucloetide motifs in plants (AC/TG)_n _and (AAG/TTC)_n _(e.g. [28]).

**Table 3:** List of iSSR- and RAPD-primers.

**type**	**denomination**	**sequence (5' → 3')**	**source**
iSSR	17898B	(CA)_6_-gT	according to [21]
	17898C	(CA)_6_-AC	
	17899	(CA)_6_-gg	
	17901B	(gT)_6_-TT	
	P02	(AAg)_6_-Cg	according to [28]

RAPD	RX13	ACgggAgCAA	random primer kits
	RX14	ACAggTgCTg	
	RY01	gTggCATCTC	
	RY13	gggTCTCggT	
	RY15	AgTCgCCCTT	
	RY16	gggCCAATgT	
	RY17	gACgTggTgA	
	RY18	gTggAgTCAg	
	RZ04	AggCTgTgCT	
	RZ05	TCCCATgCTg	
	RZ07	CCAggAggAC	
	RZ12	TCAACgggAC	
	RZ17	CCTTCCCACT	

List of primers used for PCR-amplification of mono- and polymorphic fragments within gDNA of *C. vulgaris*, their sequence, and the source according to which the sequences were selected.

Electrophoretic separation of the amplification products was performed in 23 × 25 cm 1.5% agarose gels by applying 7 V/cm for 2.5 hours. The gel contained ethidium bromide for visualization of fragments at 254 nm. Documentation was carried out with a digital imaging system (Biostep GmbH).

Reactions were repeated at least twice before fragments were used for distance calculations.

### Statistics: gel analysis and phylogenetic calculations

Gel analysis (band detection, noise reduction, size calibration, fragment matching) was performed with the Phoretix 1D Advanced software (Nonlinear Dynamics). The selection of bands derived from each primer was performed by objective criteria (e.g. thresholds for routine band detection and matching and recommendations to ensure reproducibility, e.g. the exclusion of fragments of very high and very low size). The banding data were transformed to a computable 0/1 matrix in the common [OTU × band] layout.

Phylogenetic as well as dendrogram calculations were conducted with the NTSYSpc 2.20 L software (^©^1986–2006, Applied Biostatistics Inc.). Qualitative banding values were computed using the *SimQual *module with the similarity coefficient of Dice [29] and Nei and Li [30], respectively. Subsequent UPGMA clustering was conducted within the *Sahn *module, while the module *Treeview *was used to visualize the data set as a dendrogram.

For Bootstrapping using the Dice coefficient, Winboot [31] was used (replications given in the text) which finally constructs a majority-rule consensus tree based on the *Consense *module of the PHYLIP software.

### EDV-testing by application of the tail principle

A system first published by [11] for EDV identification in lettuce and barley was adapted for *C. vulgaris *as follows. Since a priori pedigree information is unavailable for *C. vulgaris *and the application of the *pedigree principle *– a threshold selection based upon inclusion of 'identity by descent' probabilities – was not possible, we selected the *tail principle *for the identification of a threshold from a distribution of pair-wise similarities from a reference-set. The configuration of the Reference Set (25 varieties) for each Test Set (2 varieties) was adapted by using the information gained from our phylogenetic results which matches the integration of the *calibration principle*. In the context of [11], we decided not to include known EDVs in this set, e.g. mutation-derived varieties or sports, since these would only represent extremely high values within the reference-set and would complicate data interpretation. Both polymorphic and monomorphic markers were analyzed. Detached primer-wise computation of similarity values becomes applicable by assuming a genetic independence between primers and a uniform distribution of primer binding sequences throughout the genome. Arithmetic means, standard deviations, standard errors as well as its medians were calculated according to [10] and [11] in analyzing the inner- and inter-set-similarities and also to define a threshold for identifying EDVs in *Calluna*. This threshold was positioned in such a way that varieties known to be ED exceed the threshold and non-EDVs do not.

As carried out before for RAPD and iSSR values, these data were computed for their similarity values with the coefficient of Dice (Nei & Li) by NTSYSpc 2.20 L. Only in case of Dice similarity values above the threshold and non-overlapping error bars, a pair of genotypes is categorized as being essentially derived. In a case where similarity values are below the threshold or where they are above but with overlapping error bars, a pair of genotypes is categorized as not essentially derived.

## Abbreviations

AFLP: Amplified Fragment Length Polymorphism; ASSINSEL: 'International Association of Plant Breeders for the Protection of Plant Varieties'; EDV: Essentially Derived Variety/Varieties; BSA: Bundessortenamt; CPVO: Community Plant Variety Office; gDNA: genomic DNA; ISF: International Seed Federation; iSSR: inter Simple Sequence Repeats; RAPD: Randomly Amplified Polymorphic DNA; UPOV: International Union for the Protection of new Varieties of Plants; UPGMA: Unweighted Pair Group Method with Arithmetic Mean

## Authors' contributions

After methodological setup, TB carried out the complete RAPD section from laboratory work to analysis and drafted the manuscript. JK performed the complete iSSR part from laboratory work to analysis. AH designed the study and participated in drafting the manuscript.

All authors read and approved the final manuscript.

## Supplementary Material

Additional file 1 **Complete list of included varieties and genotypes, their country of origin and pedigree information where known (source is given in the last column)**. Column 3 defines the flower type either as normal, bud, multi-bracteate (multi) or filled. Sources of information are either the Bundessortenamt (BSA:doc), the appropriate website (web) of the 'The International Register of Heather Names'  or personal communications (personal contact). Click here for file
